# Dissecting the Cdc37 cochaperone code: Functional roles in chaperone-mediated stress adaptation

**DOI:** 10.1016/j.jbc.2025.110672

**Published:** 2025-09-01

**Authors:** Megan M. Mitchem, Ashley Choi, Duhita A. Mirikar, Rajlekha Deb, Andrew W. Truman

**Affiliations:** Department of Biological Sciences, The University of North Carolina at Charlotte, Charlotte, North Carolina, USA

**Keywords:** Cdc37, Hsp90, post-translational modification, yeast, stress responses, proteostasis, chaperone

## Abstract

Cell division cycle 37 (Cdc37) is a kinase-specific cochaperone that scaffolds protein kinase clients to the heat shock 90 (Hsp90) chaperone system. Although phosphorylation at residues S14 and S17 is known to regulate Cdc37 function, the broader role of phosphorylation across the protein remains unclear. To systematically investigate this, we created a “Cdc37 code collection,” a set of 46 yeast strains expressing single phosphosite mutants of Cdc37, and performed phenotypic profiling across a wide panel of environmental and chemical stressors. While canonical sites like S14 and S17 were essential for stress tolerance, 34 additional phosphomutants exhibited distinct phenotypes, often in a stress-specific manner. Notably, the mutations displayed little overlap in their stress responses, suggesting a modular and context-dependent regulation of Cdc37. Our data reveal that Cdc37 function is intricately modulated by site-specific phosphorylation, which shapes its capacity to maintain proteostasis under diverse cellular conditions. This study provides a comprehensive resource for dissecting the functional landscape of Cdc37 post-translational regulation and highlights new regulatory sites with potential relevance to chaperone–kinase network dysregulation in disease.

Maintenance of cellular proteostasis requires a suite of molecular chaperones, including heat shock proteins, Hsp70, Hsp90, and Hsp104 (1, 2, 3, 4). Cells also express numerous cochaperones that fine-tune chaperones and aid in client recruitment (1, 5). One of the most important is the cell division cycle 37 (Cdc37) cochaperone, initially identified in budding yeast during a screen for cell division cycle genes (6). This essential protein is not a direct regulator of the cell cycle like cyclins or cyclin-dependent kinases but rather acts by supporting the activity of these proteins (7, 8, 9). Compared with other cochaperones, such as the Hsp40s, Cdc37 appears to have a much narrower client specificity, particularly to kinases known to interact with Hsp90 (10). Cdc37 suppresses Hsp90 ATP hydrolysis, stabilizing the interaction between Hsp90 and its kinase substrates (11). Cdc37 comprises three distinct domains: an N-terminal domain that binds kinases, a middle domain that interacts with Hsp90, and a C-terminal domain whose function is poorly understood (12). While other chaperones and cochaperones are highly conserved between yeast and humans, Cdc37 displays a considerable variation in its C-terminal domain. This divergence may indicate a yeast-specific function for Cdc37 or reflect the broader kinase landscape present in mammalian cells. Cdc37 activity is modulated by a variety of post-translational modifications (PTMs), with phosphorylation being among the most extensively studied (7, 13). In particular, phosphorylation at serine residues S14 and S17 plays a critical regulatory role. Substitution of either site with alanine to create nonphosphorylatable mutants results in marked temperature sensitivity, accompanied by altered cell morphology and impaired interaction with a substantial subset of kinase clients (13). Notably, Cdc37 and protein kinase CKII form a reciprocal regulatory circuit: CKII phosphorylates and activates Cdc37, which in turn stabilizes CKII activity (13). Improvements in the resolution of protein mass spectrometry over recent years have permitted the discovery of many more PTMs on molecular chaperones that fine-tune activity and specificity (14, 15, 16, 17). To better understand how site-specific phosphorylation modulates Cdc37 function, we generated a comprehensive panel of yeast strains expressing single phosphomutants at each identified site. By screening these mutants against a diverse set of environmental and chemical stressors, we systematically mapped the contribution of individual phosphorylation events to stress resilience. This work defines a phosphorylation-based “cochaperone code” for Cdc37, revealing both essential regulatory nodes and stress-specific modulators of function, and offers new insight into how PTMs dynamically shape the cellular chaperone landscape.

## Results

### Creation of the Cdc37 code array

**Figure 1:**
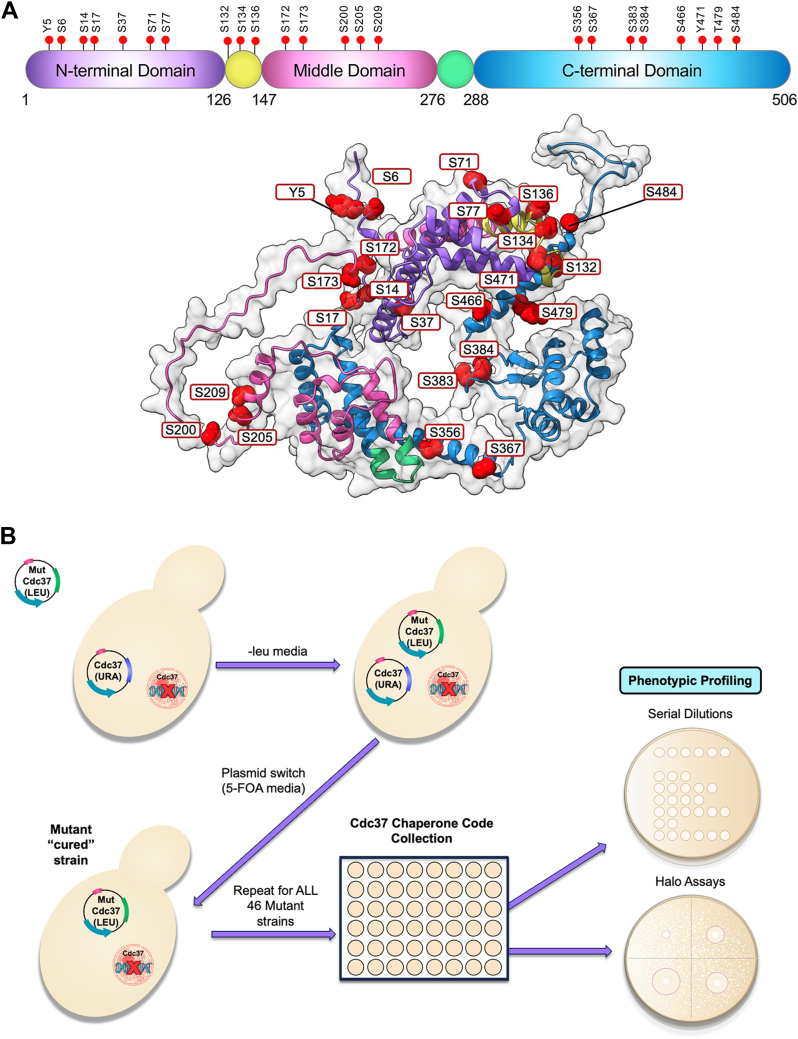
**Creation of the Cdc37 Chaperone Code Collection.***A*, experimentally determined Cdc37 phosphorylation sites from GPMDB mapped onto the AlphaFold-modeled structure of Cdc37 (AF-K7 EQA9-F1). *B*, YAT731 yeasts were transformed with *LEU2* plasmids expressing 46 phosphosite mutants of Cdc37 and then cured on 5-FOA media. The resulting 46 strains expressing Cdc37 phosphosite mutants as the sole Cdc37 in the cell were screened against a range of well-characterized cellular stresses. 5-FOA, 5-fluoro-orotic acid; Cdc37, cell division cycle 37.

Current proteomic data for yeast Cdc37 show 23 experimentally determined phosphorylation sites on Cdc37 (GPMdb, https://gpmdb.thegpm.org/). These sites are distributed across the entirety of Cdc37: seven in the N-domain, three in the interdomain linker, five in the middle domain, and eight in the C-terminal domain (Fig. 1*A*). We mapped these sites to the predicted structure for yeast Cdc37 to understand their spatial clustering (Fig. 1*A*). To gain insight into the potential importance of Cdc37 phosphorylation, we created a collection of yeast strains expressing mutations at each phosphosite to either an alanine/phenylalanine (A/F) to prevent phosphorylation or an aspartic/glutamic acid (D/E) to mimic phosphorylation (Fig. 1*B*). An *LEU2*-based plasmid expressing FLAG-tagged Cdc37 (WT and 46 respective mutants) was transformed into TM141 cdc37Δ::HIS3 (Ycplac33 *CDC37-GFP*), a yeast strain lacking the genomic copy of the *CDC37* gene but kept viable through expression of Cdc37 from a URA-based plasmid. Cells were cured on 5-fluoro-orotic acid to generate a collection of yeast that expressed single phosphosite mutations in Cdc37 as their sole Cdc37 (Fig. 1*B*). All 46 of the mutants were able to support cell viability, suggesting that these mutants retained essential Cdc37 function.

### N-terminal Cdc37 phosphorylation mutants exhibit severe sensitivity to cell wall stressors

**Figure 2:**
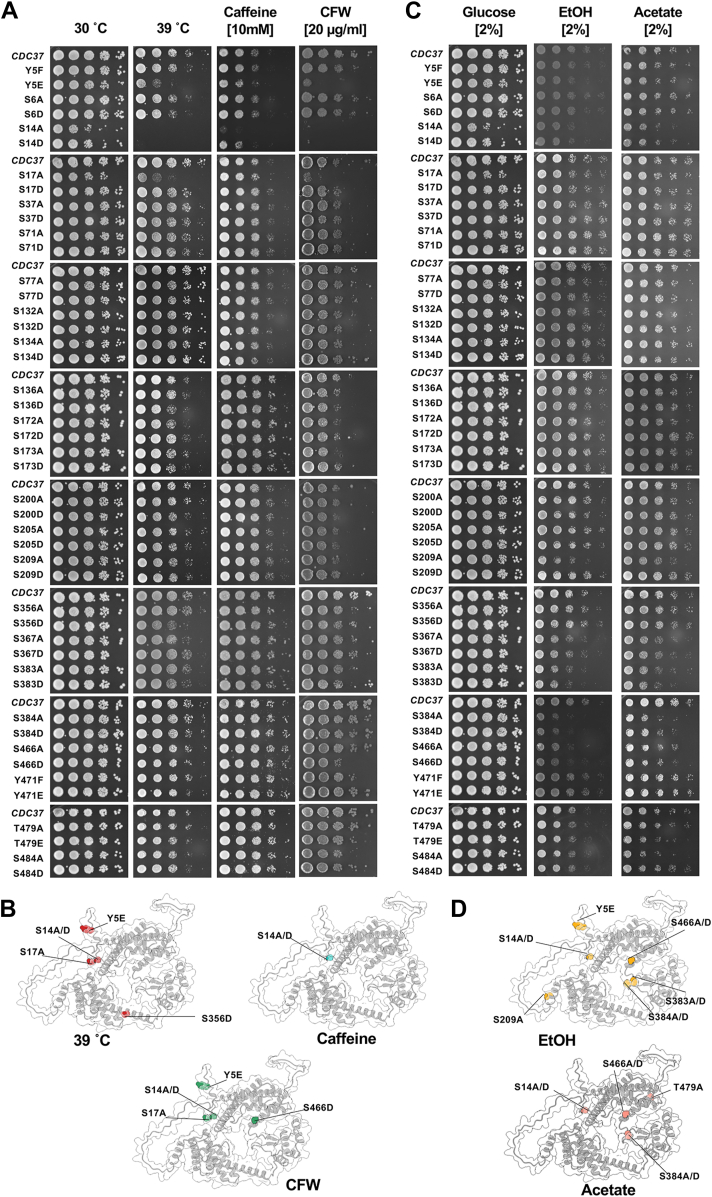
**Phenotypic fingerprinting of the Cdc37 code against cell wall and metabolic stresses.***A*, cells expressing either a nonphosphorylatable Cdc37 mutant or mimicking constant phosphorylation were grown to midlog phase and then serially diluted onto plates containing cell wall–damaging agents. Plates were incubated for 3 days at 30 °C (or 3 days at 39 °C for the high-temperature stress plates) and then photographed. *B*, phosphosites important for response to cell wall damage were mapped onto the Cdc37 structure using ChimeraX. *C*, cells were screened as in (*A*) except against nonfermentable carbon sources. *D*, phosphosites important for growth on nonfermentable carbon sources were mapped onto the Cdc37 structure using ChimeraX. The experiments in *A* and *C* were completed at the same time, and thus, the control serial dilutions shown for these are the same (*A*, column 1 and *C*, column 1). Cdc37, cell division cycle 37.

Phosphorylation at serine residues S14 and S17 has been previously implicated in the regulation of the cell wall integrity (CWI) pathway (18, 19). In addition to heat stress, several other agents are known to activate this pathway. For instance, caffeine inhibits Tor1 activity, leading to downstream activation of the CWI cascade (20), whereas calcofluor white (CFW), a chitin-binding fluorescent dye, disrupts cell wall assembly by interfering with chitin polymerization (21). To evaluate whether Cdc37 phosphorylation is required for mounting an effective CWI response, we cultured each mutant strain to midlog phase and performed serial dilutions onto plates containing either control medium (yeast extract–peptone–dextrose) or yeast extract–peptone–dextrose supplemented with caffeine (10 mM), CFW (20 μg/ml), or subjected to elevated temperature (39 ˚C). After 3 days of incubation, growth phenotypes were assessed. Consistent with prior findings, S14 and S14D mutants exhibited pronounced growth defects under all CWI stress conditions (Fig. 2, *A* and *B*). In addition, both Y5E and S17A mutants showed severe sensitivity to heat and CFW, suggesting their importance in maintaining CWI (Fig. 2, *A* and *B*). In contrast, C-terminal phosphomutants such as S356D and S466D displayed only mild sensitivity to heat and CFW, respectively, highlighting a dominant role for N-terminal phosphorylation in Cdc37-mediated CWI signaling (Fig. 2, *A* and *B*).

### C-terminal phosphorylation of Cdc37 modulates the ability of yeast to grow on nonfermentable carbon sources

Ethanol imposes cellular stress by inducing oxidative damage and compromising membrane integrity, particularly in the plasma membrane and mitochondria (22, 23). In response, yeast cells activate adaptive signaling pathways such as the high-osmolarity glycerol pathway and the heat shock response to mitigate damage (23, 24). In our screen, most Cdc37 phosphomutants did not show severe sensitivity to ethanol; however, mild growth defects were observed in strains carrying mutations Y5E, S14A, S14D, S209A, S383A, S383D, S384A, S384D, S466A, and S466D (Fig. 2, *C* and *D*). The modest nature of these phenotypes may reflect the yeast’s capacity to detoxify ethanol through alcohol dehydrogenases and antioxidant defense systems (25). Acetate, another metabolic stressor, perturbs cellular function by lowering intracellular pH and impairing mitochondrial activity (26). Several Cdc37 mutants, including S14A, S14D, S384A, S384D, S466A, S466D, and T479E, exhibited mild but consistent growth defects under acetate stress (Fig. 2, *C* and *D*). These findings suggest that phosphorylation at specific C-terminal and linker-region residues contributes to the role of Cdc37 in supporting cellular metabolism and pH homeostasis under stress conditions.

### Distinct Cdc37 phosphorylation sites modulate the response to DNA-damaging agents

**Figure 3:**
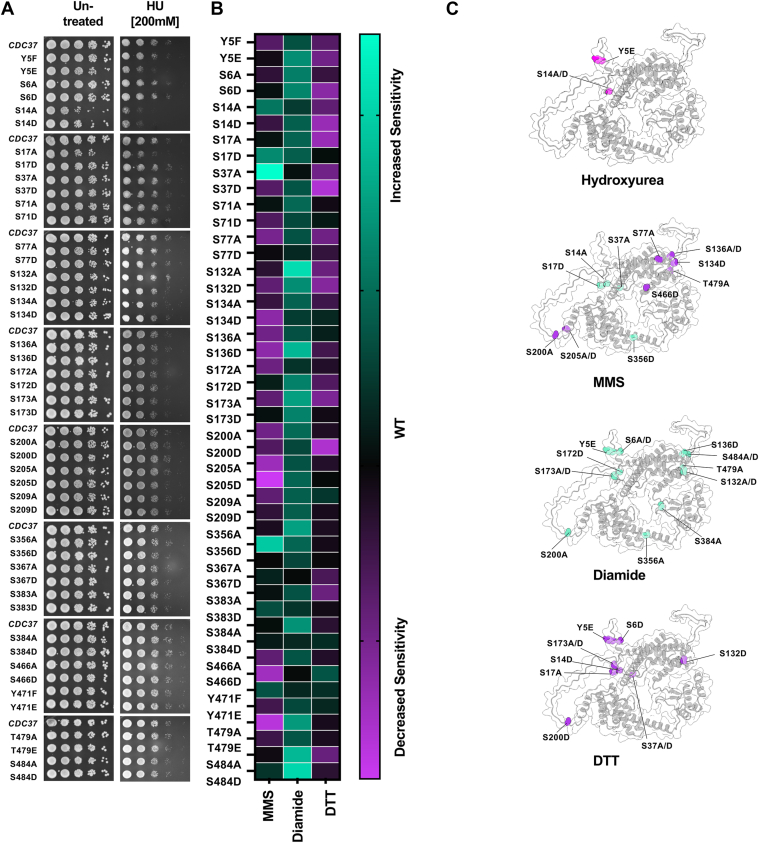
**Phenotypic fingerprinting of the Cdc37 code array against DNA-damaging/oxidative stress agents.***A*, cells expressing either a nonphosphorylatable Cdc37 mutant or mimicking constant phosphorylation were grown to midlog phase and then serially diluted onto plates containing DNA-damaging agents. Plates were incubated for 3 days at 30 °C and then photographed. This experiment was completed at the same time as the data shown in Figure 2. As such, the control dilution shown in this figure (column 1) is the same as Figure 2*A*, column 1 and 2*C*, column 1. *B*, cells expressing either a nonphosphorylatable Cdc37 mutant or mimicking constant phosphorylation were grown to midlog phase and spread onto -Leu plates using glass beads. Plates were split into quadrants. Sterile filter paper discs with indicated stress concentrations were added to three of the quadrants, and water was added to the fourth as a negative control. Plates were imaged after 2 days, and resistance to growth “halos” was measured using ImageJ software. *C*, phosphosites important for response to DNA damage/oxidative stress agents were mapped onto the Cdc37 structure using ChimeraX. *∗Green* indicates an increase in sensitivity to the stress, and *purple* indicates that the mutant cells are more resilient than WT. Cdc37, cell division cycle 37.

Molecular chaperones have increasingly been recognized as critical players in preserving genome integrity under stress (27, 28, 29, 30). Building on this, our group previously identified ribonucleotide reductase, an essential enzyme for DNA synthesis and repair, as a client of the Hsp70–Hsp90 chaperone machinery (31, 32). Given the central role of Cdc37 in kinase and client stabilization, we hypothesized that its phosphorylation state might influence the cellular response to genotoxic stress. To examine this possibility, we screened the Cdc37 Code Collection against a panel of well-characterized DNA-damaging agents: hydroxyurea (HU), a ribonucleotide reductase inhibitor that stalls DNA replication; methyl methanesulfonate (MMS), which introduces alkylation-induced DNA lesions; and diamide, a thiol-oxidizing agent that generates oxidative stress and indirectly challenges genome stability. Interestingly, the HU-sensitive mutants Y5E, S14A, S14D, and S17A heavily overlapped with those previously shown to be sensitive to cell wall stressors, suggesting a shared regulatory mechanism involving Cdc37’s N-terminal domain (Figure 2, Figure 3, *A*, *B* and 3, *A*, *B*).

Due to the instability of MMS and diamide in culture media, we employed a halo assay approach (33, 34, 35, 36, 37, 38) to assess sensitivity. In contrast to the HU results, a distinct set of mutants, including S77A, S134D, S136A, S136D, S200A, S200D, S205A, S205D, S466A, S466D, and T479A, displayed increased resistance to MMS and showed improved growth compared with WT cells (Fig. 3, *B* and *C*). These findings suggest that phosphorylation at these sites may normally restrain DNA repair capacity or modulate checkpoint activation. Conversely, S14A, S17D, S37A, and S356D mutants were hypersensitive to MMS, indicating that these residues play a role in promoting an effective DNA damage response. Diamide exerts toxicity by oxidizing cysteine residues, leading to protein misfolding and redox imbalance (39). Yeast counters this through antioxidant defense pathways. In our halo assays, 14 phosphomutants including, Y5E, S6A, S6D, S132A, S132D, S136D, S172D, S173A, S173D, S200A, S356A, S384A, T479A, and S484A/D, exhibited significantly increased sensitivity to diamide (Fig. 3, *B* and *C*). Together, these findings show that distinct phosphorylation sites on Cdc37 selectively regulate cellular responses to different forms of genomic stress, supporting a model in which phosphorylation patterns fine-tune chaperone-mediated genome maintenance.

### Cdc37 phosphorylation impacts the unfolded protein response

Approximately one-third of all proteins are folded in the endoplasmic reticulum (40, 41, 42). Several compounds, including DTT and tunicamycin, disrupt this process, leading to activation of the unfolded protein response (UPR) to restore proteostasis (43). To understand whether Cdc37 phosphorylation may impact UPR, we screened the collection against DTT and tunicamycin (Figs. 3, *B*, *C* and S1). Despite both stresses being UPR activators, the Cdc37 phosphorylation sites required for resistance varied substantially (Figs. 3, *B*, *C* and S1). Eight sites focused in the N-terminal region (Y5E, S6D, S14D, S17A, S37A, S37D, S132D, S173A, and S200D) regulated the response to DTT, with only two mutants, Y5E and S14A, impacting the response to tunicamycin (Figs. 3, *B*, *C* and S1).

### A novel Cdc37 phosphorylation site impacts TOR signaling

**Figure 4:**
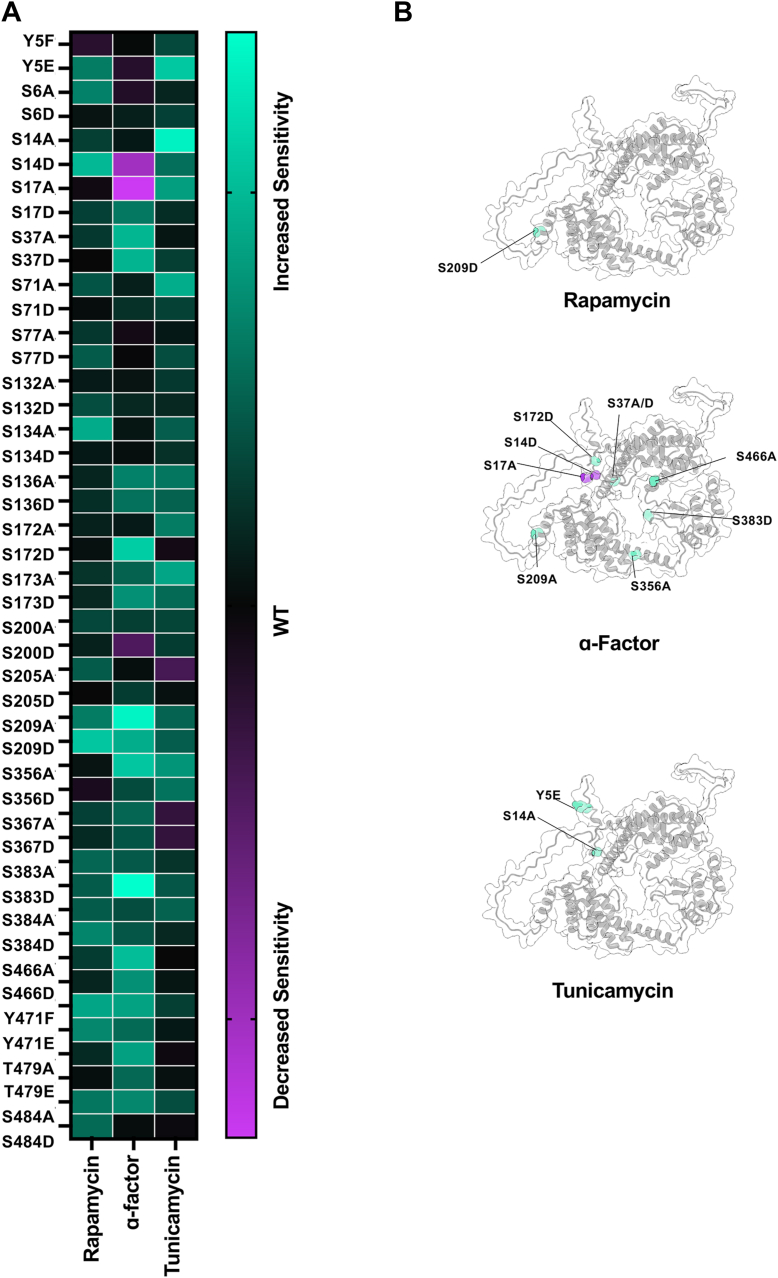
**Cdc37 mutants show a unique pattern of response to stressors.***A*, halo assays of Cdc37-mutant strains were completed as in 3*B*. *B*, sites showing more than a 20% shift from the WT phenotype were mapped onto the Cdc37-predicted structure using ChimeraX. ∗*Green* indicates an increase in sensitivity to the stress, and *purple* indicates that the mutant cells are more resilient than WT. Cdc37, cell division cycle 37.

Pharmacological or genetic inhibition of Hsp90 or Tel2 destabilizes TOR complexes and thus renders yeast hypersensitive to rapamycin (44). To determine whether Cdc37 phosphorylation may impact TOR signaling, we screened the Cdc37 collections against rapamycin (Figs. 4, *A*, *B* and S1). In contrast to the other stresses tested, only a single Cdc37 mutant, S209D, was sensitive to rapamycin (Figs. 4, *A*, *B* and S1).

### A spectrum of Cdc37 phosphorylation sites alters pheromone response signaling

Previous studies have identified a role for Cdc37 in Ste11 kinase function and pheromone-mediated cell cycle arrest (45). Upon screening the code collection against arrest in response to alpha factor, we observed a range of responses (Figs. 4, *A*, *B* and S1). S37A, S37D, S172D, S209A, S356A, S383D, and S466A became sensitized to alpha factor, whereas S14D and S17A became pheromone resistant (Figs. 4, *A*, *B* and S1).

### A subset of Cdc37 mutants displays altered protein levels

**Figure S2:**
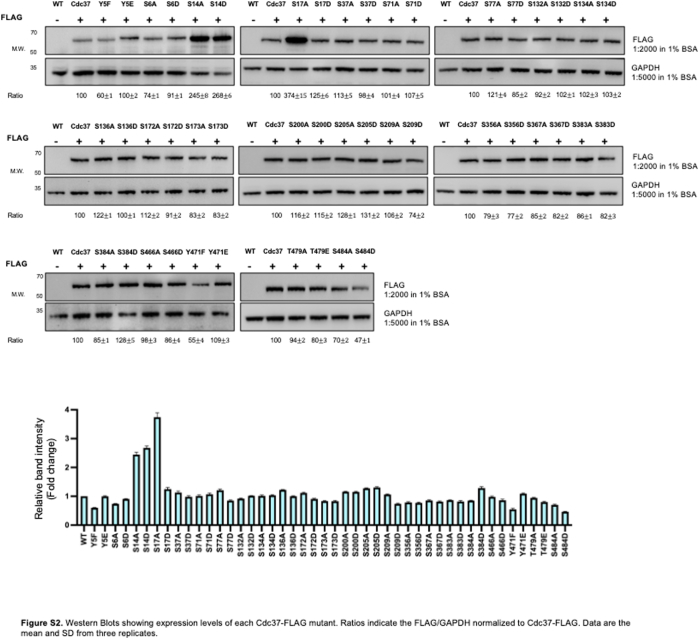


We considered the possibility that the phenotypic effects of our Cdc37 mutants may be a result of altered steady-state levels. We assessed the abundance of FLAG-Cdc37 in all 46 mutants *via* Western blotting (Fig. S2). Only S14A, S14D, and S17A mutants displayed a significant increase in Cdc37 levels, with the other mutants expressing at levels comparable to the WT (Fig. S2).

## Discussion

**Figure 5:**
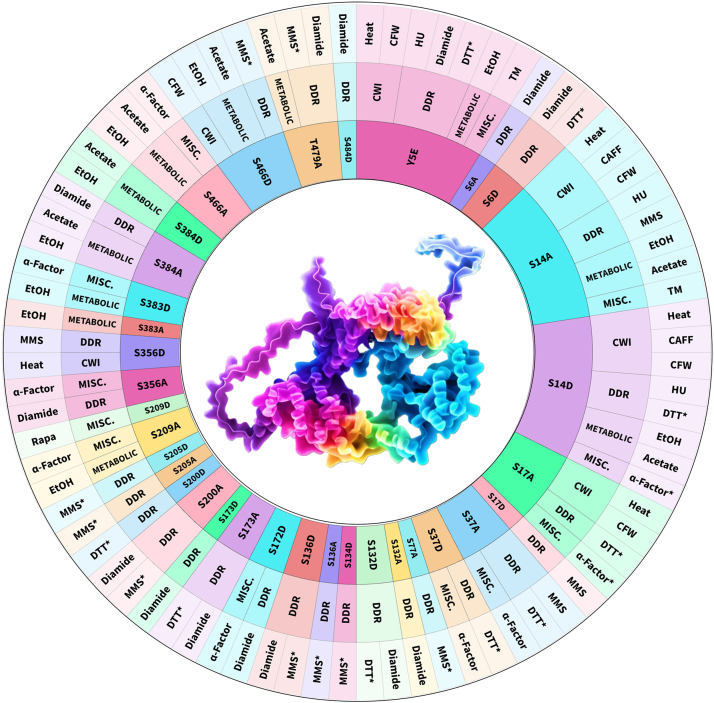
**Summary: Phenotypic profiling of the Cdc37 code array reveals PTM master regulators *versus* fine-tuners of Cdc37 activity.***Asterisks* (∗) indicate the mutant strain is more resilient than WT. Cdc37, cell division cycle 37; PTM, post-translational modification.

This study introduces the Cdc37 Code Collection as a systematic and powerful resource for dissecting the phosphorylation-based regulation of the Hsp90 cochaperone Cdc37. By engineering a comprehensive panel of phospho-null and phosphomimetic mutations at 23 known or predicted phosphorylation sites across the Cdc37 protein, we created a modular toolkit that enables detailed functional analysis of cochaperone regulation across a broad array of cellular stresses. This collection not only confirms the importance of canonical sites but also unveils a previously unappreciated landscape of site-specific, context-dependent phosphorylation events that modulate Cdc37 activity (Fig. 5). The spatial distribution of phosphosites across Cdc37’s three domains offers valuable insight into the functional roles of these modifications. The N-terminal domain, which mediates kinase client binding, harbors several high-impact regulatory residues, including Y5, S14, and S17. Mutations at these sites conferred the strongest and broadest sensitivity phenotypes across multiple stressors, including heat, caffeine, CFW, HU, and response to pheromone, highlighting their roles as master regulators of Cdc37 function (Fig. 5). These residues likely influence both direct kinase binding and conformational states critical for client delivery. The overlap between mutants sensitive to cell wall stresses and HU may be explained by the fact that the response to these stresses relies on the activity of the Mpk1 mitogen-activated protein kinase, a well-established client of the Hsp90–Cdc37 system (36). In contrast, mutations in the middle and C-terminal domains, such as S356, S383, S384, and S466, generally resulted in milder, stress-specific phenotypes. These findings suggest that many of these sites act as fine-tuners of Cdc37 function, optimizing the chaperone–client interaction for particular environmental or metabolic contexts, such as during oxidative stress or under pH-altering conditions like acetate exposure. The collection also enables a distinction between previously characterized regulatory sites (*e.g.*, S14 and S17) and newly discovered functional sites. Our data show that while known sites are essential for general chaperone activity, several newly identified residues, such as S200, S205, S384, and T479, exhibit regulation of discrete responses, such as DNA damage resistance or ethanol tolerance. For instance, S384D and T479A mutants showed altered responses to acetate and diamide, implicating these residues in metabolic stress responses and redox regulation. During the course of our study, we considered the possibility that the phenotypes we observed may be the effect of altered Cdc37 steady-state levels. Our immunoblots revealed only differences in S14A, S14D, and S17A strains, which expressed Cdc37 at higher levels than WT. This has been noted in previous studies, and it is likely that the increased abundance is a feedback response to compensate for reduced Cdc37 activity in these mutants (46). It is interesting to note that mutation of some sites, such as S14, produces a similar effect when mutated to either a phosphomimic or a phosphomutant amino acid. It may be that the mimic substitutions in this case cannot fully match the negative charge of a phosphorylated residue or that the dynamic switching between phosphorylated and nonphosphorylated states is key for functionality. We hope to investigate this in future, more targeted studies.

These discoveries expand the current model of Cdc37 regulation beyond a few static modifications to a dynamic, multilayered phosphorylation code. Moreover, the observed nonoverlapping stress sensitivities among many mutants argue against a purely hierarchical phosphorylation model. Instead, they support a modular, combinatorial logic in which individual sites confer specialized responsiveness to different cellular cues. This aligns with emerging models of the broader chaperone code, in which PTMs dynamically sculpt chaperone interactions and client prioritization under changing physiological conditions. The implications for the chaperone field are substantial. The Cdc37 Code Collection offers an experimental roadmap for analogous efforts targeting other cochaperones or chaperones with high PTM density, such as Hsp70 or Hsp90. As with histone and transcription factor codes, deciphering the “cochaperone code” opens avenues to predict and manipulate protein folding networks in health and disease. This is particularly relevant in the context of cancer, where Cdc37 and its kinase clients are frequently dysregulated, and in neurodegenerative disease, where proteostasis networks are under chronic stress. Future work should leverage this code array to map kinase-specific dependencies on Cdc37 phosphorylation status, define upstream kinases and phosphatases controlling these events, and extend similar logic to human Cdc37. In addition, quantitative proteomic and interactomic studies could assess how individual phosphovariants alter client repertoire and cochaperone complex dynamics. By offering both a functional atlas and a modular genetic toolkit, this study lays a foundation for future efforts by the chaperone community to decode the phosphorylation-driven regulation of molecular chaperones and their networks.

## Experimental procedures

### Yeast strains and growth conditions

Yeast cultures were grown in SD (0.67% yeast nitrogen base without amino acids and carbohydrates, 2% glucose) supplemented with the appropriate nutrients to select for plasmids and tagged genes. *Escherichia coli* DH5α was used to propagate all plasmids. *E. coli* cells were cultured in Luria broth medium (1% Bacto tryptone, 0.5% Bacto yeast extract, and 1% NaCl) and transformed to ampicillin or kanamycin resistance by standard methods.

### Bacterial strains and growth conditions

*E. coli* DH5α was used to propagate all plasmids. The BL-21 strain was used for protein expression. *E. coli* cells were cultured in Luria broth (1% tryptone, 0.5% yeast extract, and 1% NaCl) and transformed to carbenicillin resistance by standard methods. Plasmids are listed in Table S3.

### Plasmid construction

pYCP-LEU2-Cdc37 promoter-CDC37-3xFLAG was constructed by VectorBuilder (full sequence available on request). This plasmid expresses C-terminally 3xFLAG-tagged Cdc37 from the native Cdc37 promoter. All Cdc37 mutant plasmids were generated by GenScript. All plasmids were fully sequenced *via* Plasmidsaurus (sequence available on request).

### Creating the Cdc37 Code Collection

A yeast strain with the*CDC37* gene deleted and functionally complemented by Cdc37 expressed from a URA3-marked centromeric plasmid (cdc37Δ::HIS3 YCP33-Cdc37-GFP) was transformed with a LEU2-based plasmid that drives the expression of either WT FLAG-tagged Cdc37 or a phosphosite mutant for each of the 23 Cdc37 phosphosites. The URA3 plasmid was evicted on 5-fluoro-orotic acid media to yield strains where the mutant Cdc37 was the only one present in the cell. A list of the strains can be found in Table S3.

### Growth assays

For serial dilutions, yeast cells were grown to midlog phase, then 10-fold serially diluted, and subsequently plated onto the appropriate media using a 48-pin replica-plating tool. Images of plates were taken after 3 days at 30 C. The serial dilutions shown in Figures 2 and 3 were completed at the same time. To test a wide range of stressors, dilutions were performed on plates containing either EtOH (2%), acetate (2%), HU (200 mM), CFW (20 μg/ml), caffeine (10 mM), or heat-shocked cells at 37 C and 39 C. For growth curves, yeast cells were grown to midlog phase, and absorbance was measured at 600 nM for the indicated times. The colony growth of each of these was compared with that under unstressed conditions to evaluate phenotypic differences in at least triplicate.

### Yeast halo assay

For halo assay, yeast cells were grown to midlog phase. The following day, the culture was diluted at 1:1000, and 150 μl of cells were spread onto a -Leu plate. To test for stressors, 2 mg/ml tunicamycin, 1 M DDT, 3 pM rapamycin, 0.75 M diamide, 5% (v/v) MMS, and 5 μg/ml synthetic α-factor peptide (WHWLQLKPGQPNleY) were spotted onto circular filter paper and placed onto the aforementioned media. The plate was incubated for 2 days at 30 °C and then photographed. Measurements were taken using ImageJ. The data were then analyzed using GraphPad Prism 10 (GraphPad Software, Inc).

### Immunoblotting

To measure Cdc37 expression in the Cdc37-FLAG mutants, cells were grown to midlog phase, and protein extracts were made as described (46). Approximately 20 μg of protein was separated by 4% to 12% NuPAGE SDS-PAGE (Thermo Fisher Scientific). Proteins were detected using the primary antibodies (Table S3) with the following dilutions: anti-GAPDH (1:5000 dilution) and anti-FLAG tag (1:2000 dilution) in 1% bovine serum albumin. Membranes were washed with Tris-buffered saline–Tween 20 (0.2%) and incubated with the corresponding secondary antibody, anti-mouse immunoglobulin G–horseradish peroxidase (1:5000 dilution). After treatment with SuperSignal West Pico Chemiluminescent Substrate (Thermo Fisher Scientific), blots were imaged on a ChemiDoc MP imaging system (Bio-Rad).

### General data analyses

Halos from the halo assays were quantified using ImageJ image analysis software (https://imagej.net/ij/). Data processing and analyses were performed *via* GraphPad Prism (version 10.4.2). The diagram in Figure 5 was created using Flourish (https://app.flourish.studio/visualisation/23492773/edit). Cdc37 abundance was quantified using ImageLab software (https://www.bio-rad.com/en-us/product/image-lab-software?ID=KRE6P5E8Z). Results were normalized against GAPDH in each strain, and the ratios in WT Cdc37-FLAG were defined as onefold. Data represented are the mean and SD from three replicates.

### Protein visualization

The AlphaFold-predicted structure of Cdc37 (AF-K7 EQA9-F1) was rendered using ChimeraX software (https://www.cgl.ucsf.edu/chimera/) (Figure 1, Figure 2, Figure 3, Figure 4*B* and 5). The rainbow overlay on Figure 5 was added using Dzine.

## Data availability

All data are available in the included figures and supporting information.

## Supporting information

This article contains supporting information.

## Conflict of interest

The authors declare that they have no conflicts of interest with the contents of this article.

## References

[1] Omkar S., Shrader C., Hoskins J.R., Kline J.T., Mitchem M.M., Nitika (2024). Acetylation of the yeast Hsp40 chaperone protein Ydj1 fine-tunes proteostasis and translational fidelity. bioRxiv.

[2] Girstmair H., Tippel F., Lopez A., Tych K., Stein F., Haberkant P. (2019). The Hsp90 isoforms from S. cerevisiae differ in structure, function and client range. Nat. Commun..

[3] Wickramaratne A.C., Liao J.-Y., Doyle S.M., Hoskins J.R., Puller G., Scott M.L. (2023). J-domain proteins form binary complexes with Hsp90 and ternary complexes with Hsp90 and Hsp70. J. Mol. Biol..

[4] Zhao X., Stanford K., Ahearn J., Masison D.C., Greene L.E. (2023). Hsp70 binding to the N-terminal domain of Hsp104 regulates [PSI+] curing by Hsp104 overexpression. Mol. Cell. Biol..

[5] Rosenzweig R., Nillegoda N.B., Mayer M.P., Bukau B. (2019). The Hsp70 chaperone network. Nat. Rev. Mol. Cell Biol..

[6] Reed S.I. (1980). The selection of amber mutations in genes required for completion of start, the controlling event of the cell division cycle of S. cerevisiae. Genetics.

[7] Ren M., Santhanam A., Lee P., Caplan A., Garrett S. (2007). Alteration of the protein kinase binding domain enhances function of the Saccharomyces cerevisiae molecular chaperone Cdc37. Eukaryot. Cell..

[8] Oberoi J., Guiu X.A., Outwin E.A., Schellenberger P., Roumeliotis T.I., Choudhary J.S. (2022). HSP90-CDC37-PP5 forms a structural platform for kinase dephosphorylation. Nat. Commun..

[9] Pearl L.H. (2005). Hsp90 and Cdc37 -- a chaperone cancer conspiracy. Curr. Opin. Genet. Dev..

[10] Taipale M., Krykbaeva I., Koeva M., Kayatekin C., Westover K.D., Karras G.I. (2012). Quantitative analysis of HSP90-client interactions reveals principles of substrate recognition. Cell.

[11] Cox M.B., Johnson J.L. (2011). The role of p23, hop, immunophilins, and other co-chaperones in regulating Hsp90 function. Methods Mol. Biol..

[12] Calderwood S.K., Blatch G.L., Edkins A.L. (2015). The Networking of Chaperones by Co-chaperones: Control of Cellular Protein Homeostasis.

[13] Bandhakavi S., McCann R.O., Hanna D.E., Glover C.V.C. (2003). A positive feedback loop between protein kinase CKII and Cdc37 promotes the activity of multiple protein kinases. J. Biol. Chem..

[14] Backe S.J., Sager R.A., Woodford M.R., Makedon A.M., Mollapour M. (2020). Post-translational modifications of Hsp90 and translating the chaperone code. J. Biol. Chem..

[15] Mitchem M.M., Shrader C., Abedi E., Truman A.W. (2023). Novel insights into the post-translational modifications of Ydj1/DNAJA1 co-chaperones. Cell Stress Chaperones.

[16] Nitika, Truman A.W. (2017). Cracking the chaperone code: cellular roles for Hsp70 phosphorylation. Trends Biochem. Sci..

[17] Backe S.J., Woodford M.R., Ahanin E., Sager R.A., Bourboulia D., Mollapour M. (2023). Impact of Co-chaperones and posttranslational modifications toward Hsp90 drug sensitivity. Subcell. Biochem..

[18] Kamada Y., Jung U.S., Piotrowski J., Levin D.E. (1995). The protein kinase C-activated MAP kinase pathway of Saccharomyces cerevisiae mediates a novel aspect of the heat shock response. Genes Dev..

[19] González-Rubio G., Martín H., Molina M. (2023). The mitogen-activated protein kinase Slt2 promotes asymmetric cell cycle arrest and reduces TORC1-Sch9 signaling in yeast lacking the protein phosphatase Ptc1. Microbiol. Spectr..

[20] Reinke A., Chen J.C.-Y., Aronova S., Powers T. (2006). Caffeine targets TOR complex I and provides evidence for a regulatory link between the FRB and kinase domains of Tor1p. J. Biol. Chem..

[21] García-Rodriguez L.J., Durán A., Roncero C. (2000). Calcofluor antifungal action depends on chitin and a functional high-osmolarity glycerol response (HOG) pathway: evidence for a physiological role of the Saccharomyces cerevisiae HOG pathway under noninducing conditions. J. Bacteriol..

[22] Martinez-Ortiz C., Carrillo-Garmendia A., Correa-Romero B.F., Canizal-García M., González-Hernández J.C., Regalado-Gonzalez C. (2019). SNF1 controls the glycolytic flux and mitochondrial respiration. Yeast.

[23] Piper P.W. (1995). The heat shock and ethanol stress responses of yeast exhibit extensive similarity and functional overlap. FEMS Microbiol. Lett..

[24] Udom N., Chansongkrow P., Charoensawan V., Auesukaree C. (2019). Coordination of the cell wall integrity and high-osmolarity glycerol pathways in response to ethanol stress in Saccharomyces cerevisiae. Appl. Environ. Microbiol..

[25] Sahana G.R., Balasubramanian B., Joseph K.S., Pappuswamy M., Liu W.-C., Meyyazhagan A. (2024). A review on ethanol tolerance mechanisms in yeast: current knowledge in biotechnological applications and future directions. Process. Biochem..

[26] Moffett J.R., Puthillathu N., Vengilote R., Jaworski D.M., Namboodiri A.M. (2020). Acetate revisited: a key biomolecule at the nexus of metabolism, epigenetics and oncogenesis-part 1: acetyl-coa, acetogenesis and acyl-CoA short-chain synthetases. Front. Physiol..

[27] Wang Y., Fernandez A., Pei X., Liu B., Shen L., Yan Y. (2024). EGFR-mediated HSP70 phosphorylation facilitates PCNA association with chromatin and DNA replication. Nucleic Acids Res..

[28] Omkar S., Wani T.H., Zheng B., Mitchem M.M., Truman A.W. (2022). The APE2 exonuclease is a client of the Hsp70-Hsp90 axis in yeast and mammalian cells. Biomolecules.

[29] Fang Q., Inanc B., Schamus S., Wang X.-H., Wei L., Brown A.R. (2014). HSP90 regulates DNA repair via the interaction between XRCC1 and DNA polymerase β. Nat. Commun..

[30] Pennisi R., Ascenzi P., di Masi A. (2015). Hsp90: a new player in DNA repair?. Biomolecules.

[31] Koç A., Wheeler L.J., Mathews C.K., Merrill G.F. (2004). Hydroxyurea arrests DNA replication by a mechanism that preserves basal dNTP pools. J. Biol. Chem..

[32] Sluder I.T., Nitika, Knighton L.E., Truman A.W. (2018). The Hsp70 co-chaperone Ydj1/HDJ2 regulates ribonucleotide reductase activity. Plos Genet..

[33] Truman A.W., Kristjansdottir K., Wolfgeher D., Hasin N., Polier S., Zhang H. (2012). CDK-dependent Hsp70 Phosphorylation controls G1 cyclin abundance and cell-cycle progression. Cell.

[34] Truman A.W., Millson S.H., Nuttall J.M., Mollapour M., Prodromou C., Piper P.W. (2007). In the yeast heat shock response, Hsf1-directed induction of Hsp90 facilitates the activation of the Slt2 (Mpk1) mitogen-activated protein kinase required for cell integrity. Eukaryot. Cell.

[35] Millson S.H., Truman A.W., Piper P.W. (2022). Hsp90 and phosphorylation of the Slt2(Mpk1) MAP kinase activation loop are essential for catalytic, but not non-catalytic, Slt2-mediated transcription in yeast. Cell Stress Chaperones.

[36] Hawle P., Horst D., Bebelman J.P., Yang X.X., Siderius M., van der Vies S.M. (2007). Cdc37p is required for stress-induced high-osmolarity glycerol and protein kinase C mitogen-activated protein kinase pathway functionality by interaction with Hog1p and Slt2p (Mpk1p). Eukaryot. Cell..

[37] Millson S.H., Truman A.W., King V., Prodromou C., Pearl L.H., Piper P.W. (2005). A two-hybrid screen of the yeast proteome for Hsp90 interactors uncovers a novel Hsp90 chaperone requirement in the activity of a stress-activated mitogen-activated protein kinase, Slt2p (Mpk1p). Eukaryot. Cell..

[38] Truman A.W., Millson S.H., Nuttall J.M., King V., Mollapour M., Prodromou C. (2006). Expressed in the yeast Saccharomyces cerevisiae, human ERK5 is a client of the Hsp90 chaperone that complements loss of the Slt2p (Mpk1p) cell integrity stress-activated protein kinase. Eukaryot. Cell.

[39] Wemmie J.A., Steggerda S.M., Moye-Rowley W.S. (1997). The Saccharomyces cerevisiae AP-1 protein discriminates between oxidative stress elicited by the oxidants H2O2 and diamide. J. Biol. Chem..

[40] Jämsä E., Simonen M., Makarow M. (1994). Selective retention of secretory proteins in the yeast endoplasmic reticulum by treatment of cells with a reducing agent. Yeast.

[41] Craig E.A., Gambill B.D., Nelson R.J. (1993). Heat shock proteins: molecular chaperones of protein biogenesis. Microbiol. Rev..

[42] Luo Y., Zhong J.-J., Xiao H. (2025). Mechanism and engineering of endoplasmic reticulum-localized membrane protein folding in Saccharomyces cerevisiae. Metab. Eng..

[43] Matabishi-Bibi L., Challal D., Barucco M., Libri D., Babour A. (2022). Termination of the unfolded protein response is guided by ER stress-induced HAC1 mRNA nuclear retention. Nat. Commun..

[44] Millson S.H., Piper P.W. (2014). Insights from yeast into whether the inhibition of heat shock transcription factor (Hsf1) by rapamycin can prevent the Hsf1 activation that results from treatment with an Hsp90 inhibitor. Oncotarget.

[45] Abbas-Terki T., Donzé O., Picard D. (2000). The molecular chaperone Cdc37 is required for Ste11 function and pheromone-induced cell cycle arrest. FEBS Lett..

[46] Millson S., van Oosten-Hawle P., Alkuriji M.A., Truman A., Siderius M., Piper P.W. (2014). Cdc37 engages in stable, S14A mutation-reinforced association with the most atypical member of the yeast kinome, Cdk-activating kinase (Cak1). Cell Stress Chaperones.

