# Supplementary figures and images for: Dissecting the Cdc37 cochaperone code: Functional roles in chaperone-mediated stress adaptation
Source: J Biol Chem. 2025 Sep 1;301(10):110672. doi: 10.1016/j.jbc.2025.110672 (PMC12506479; doi:10.1016/j.jbc.2025.110672)

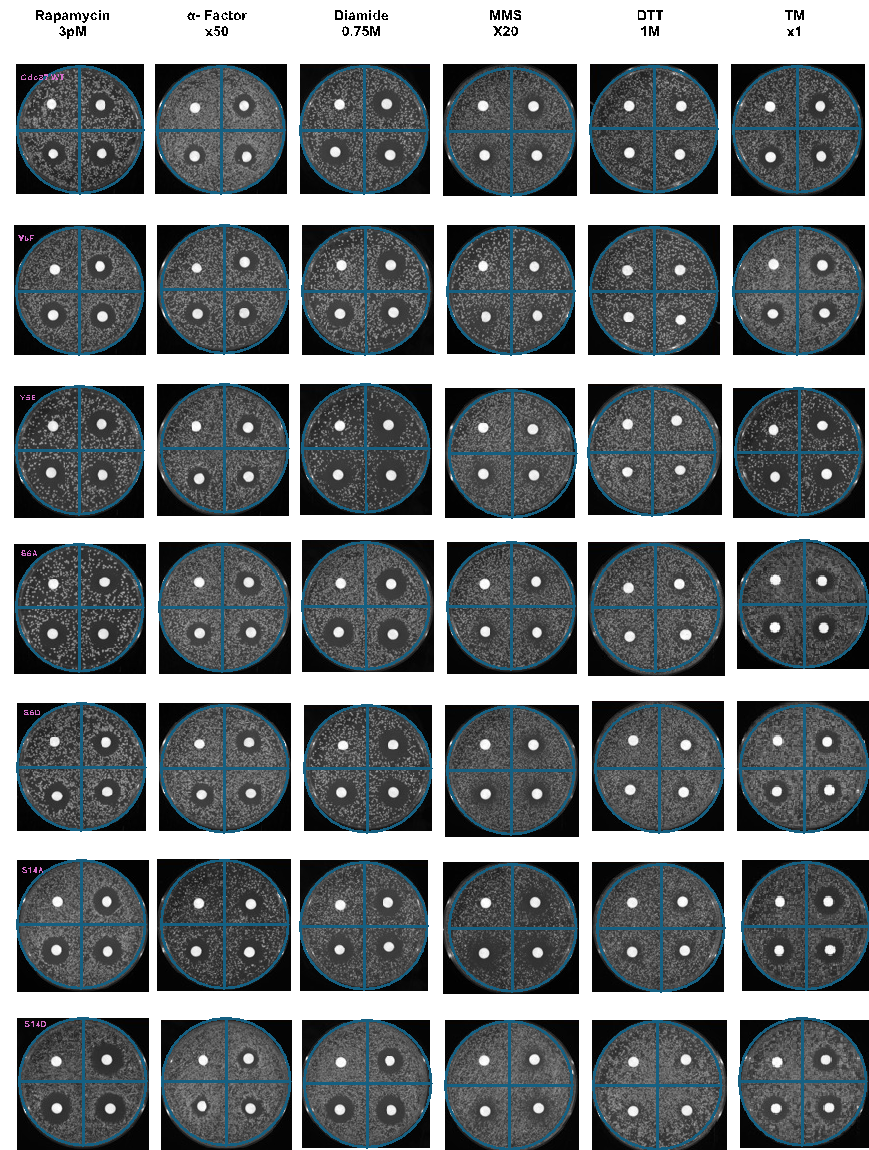

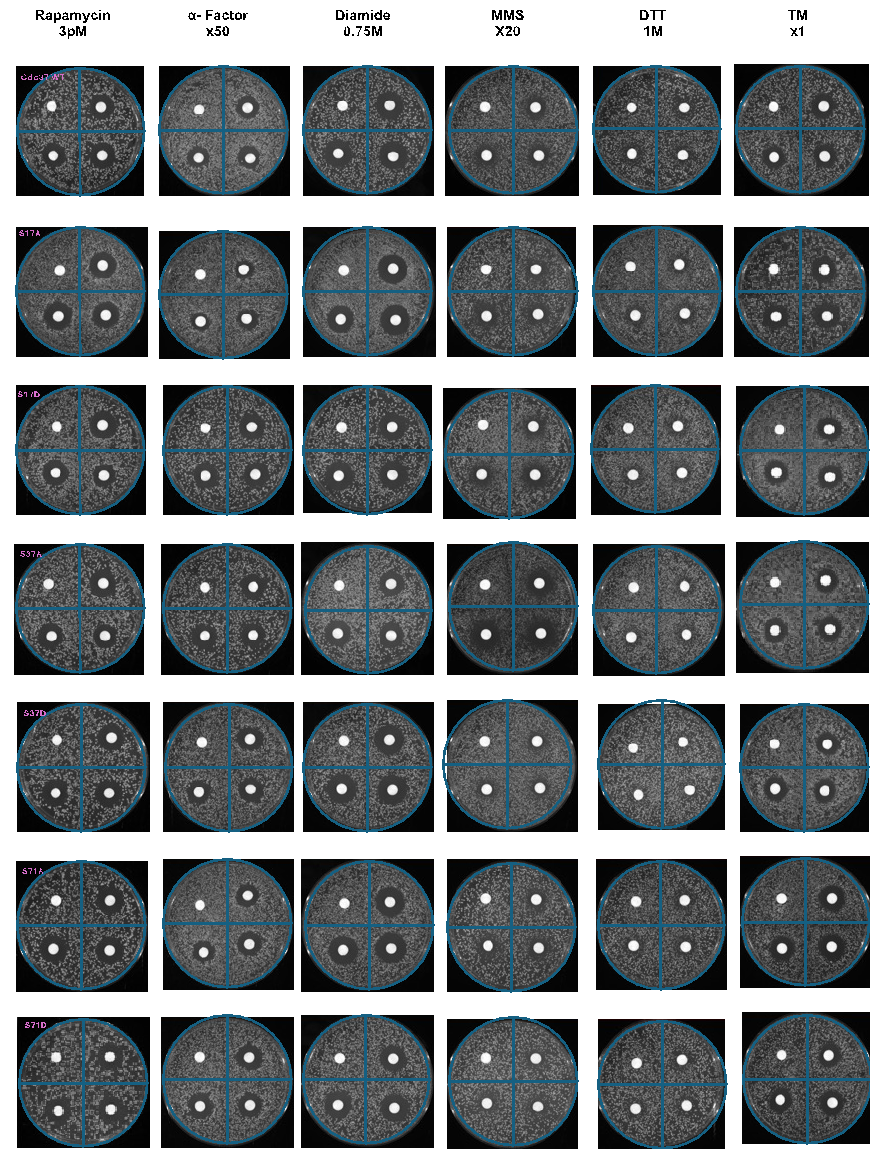

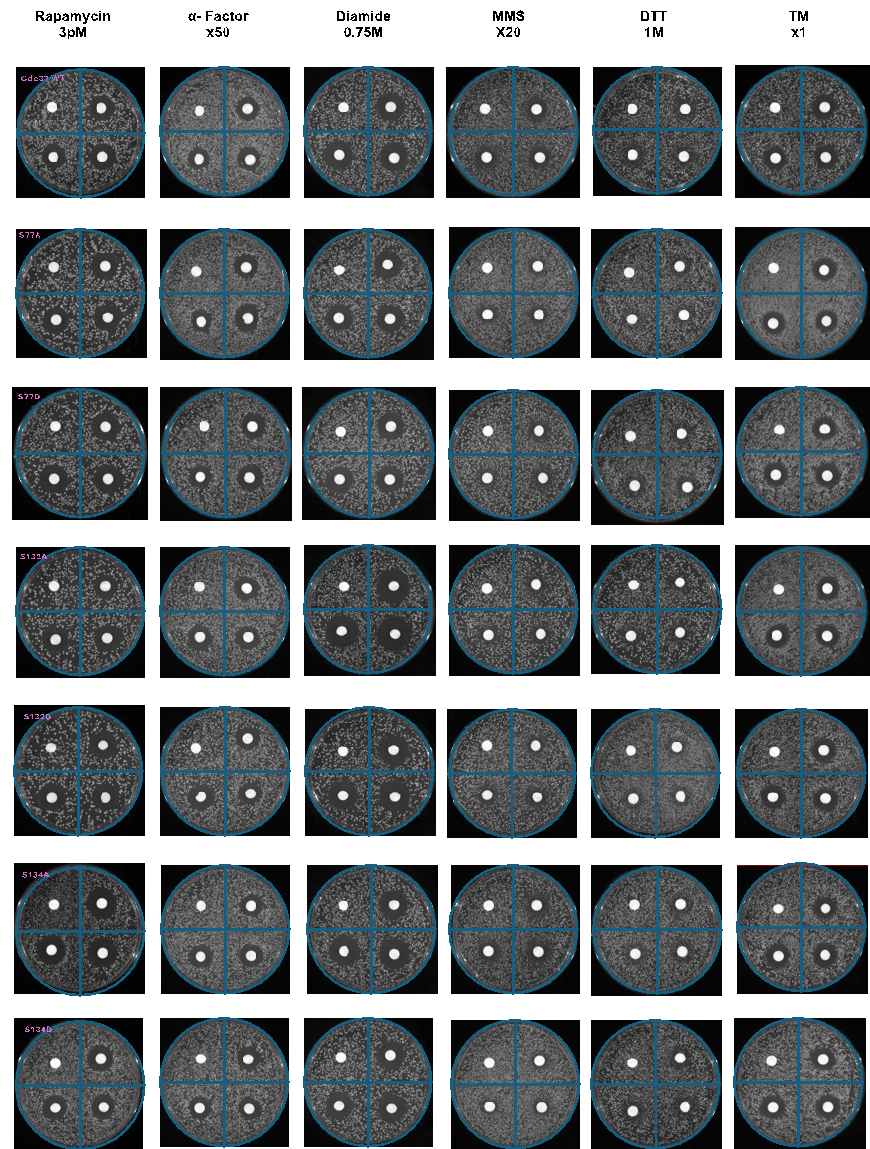

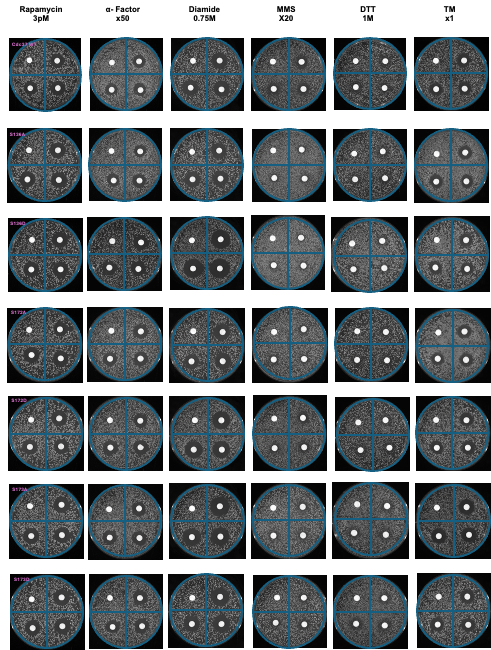

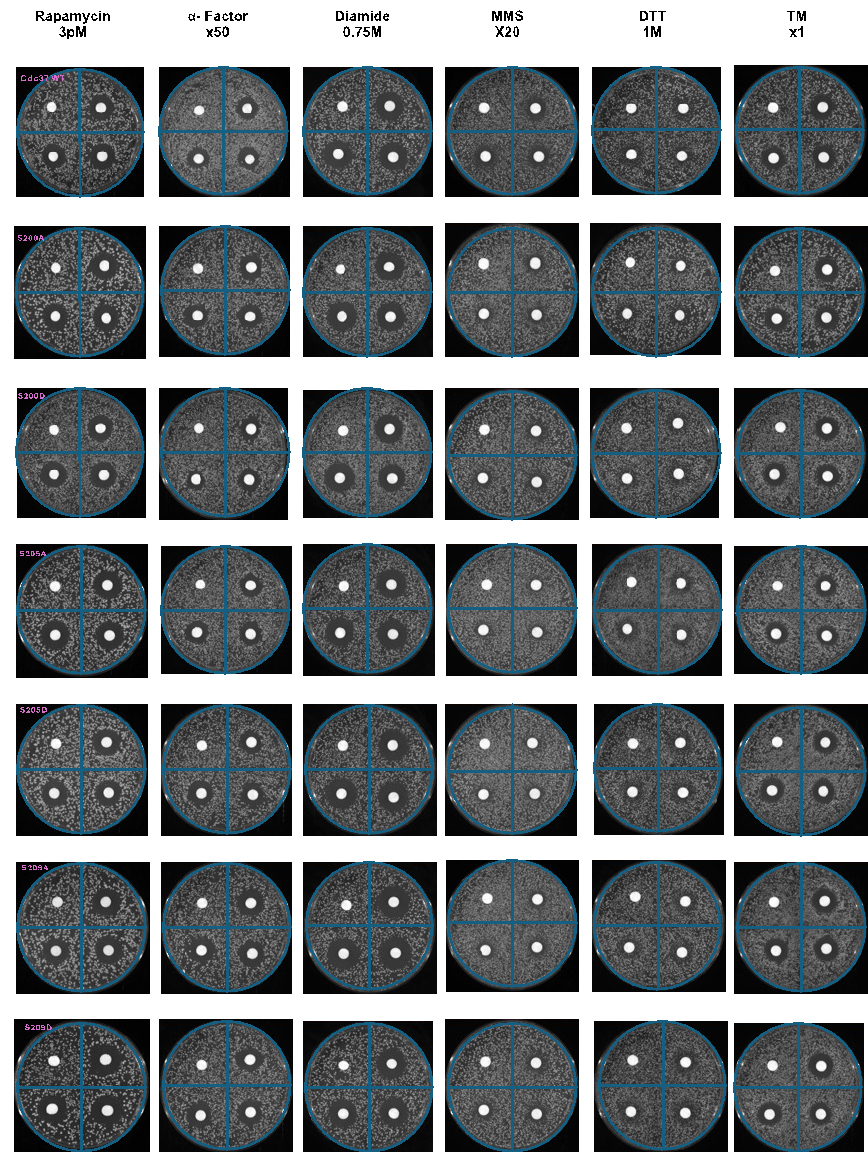

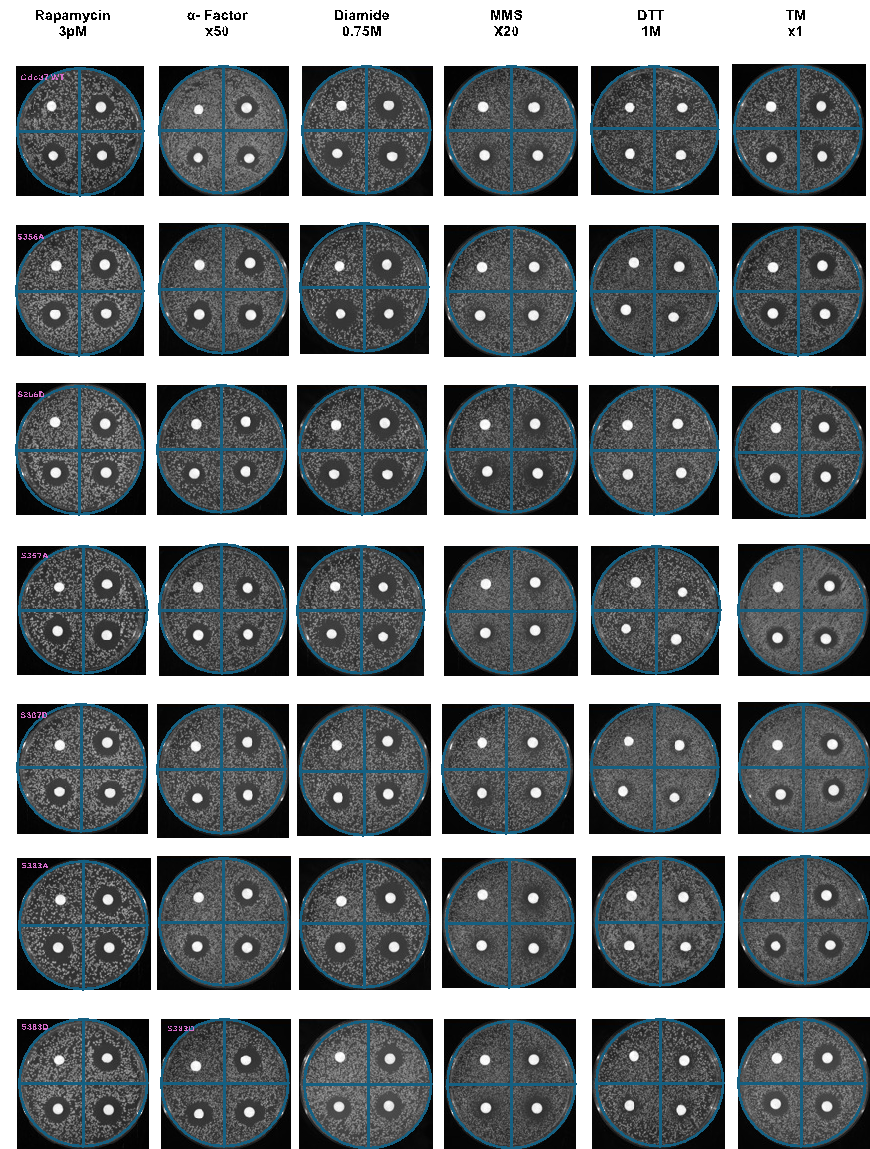

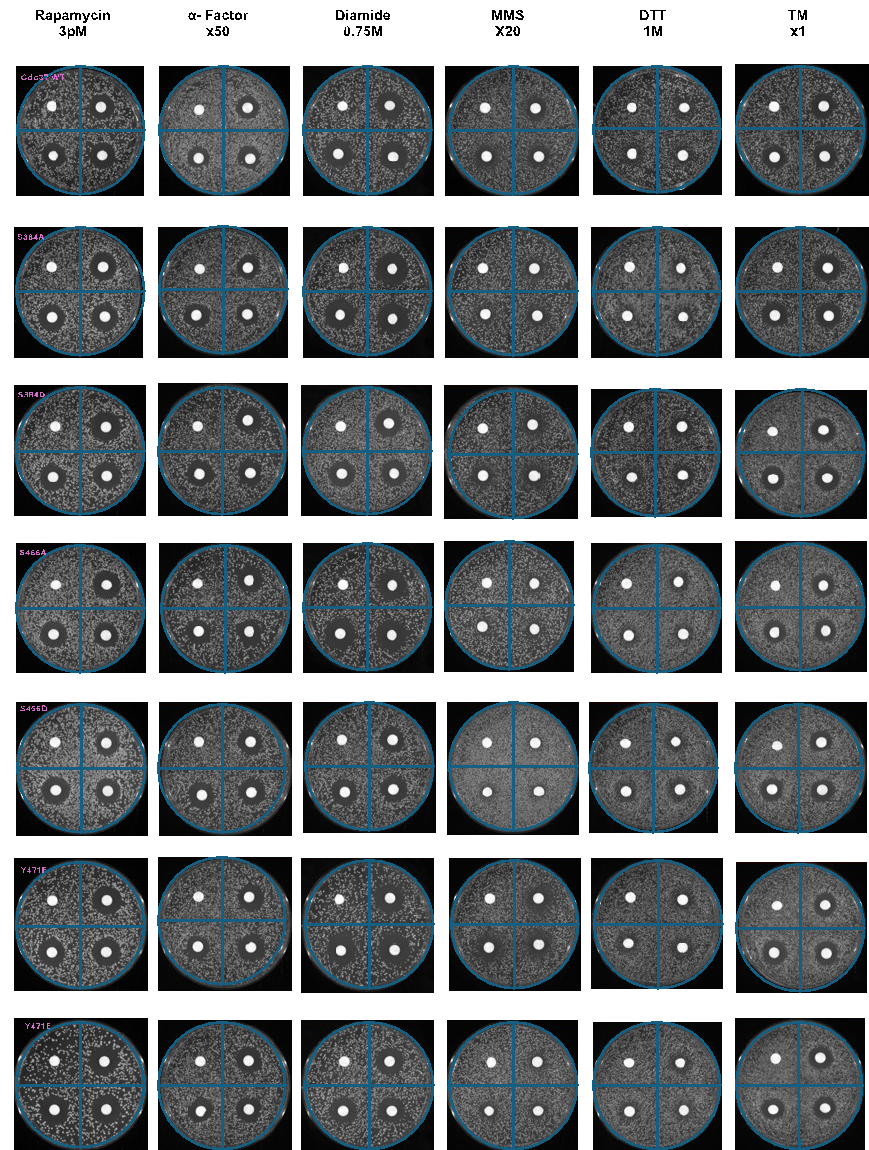

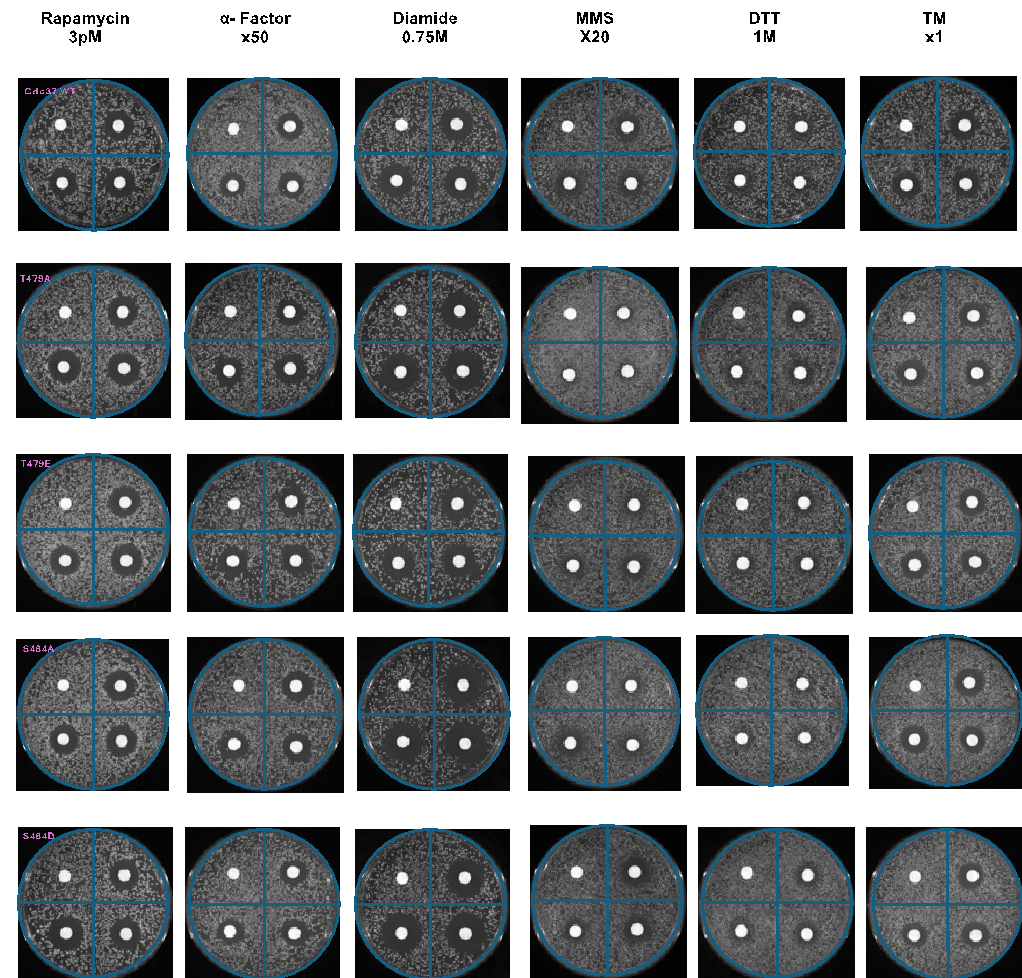


**Figure S1.** Raw images of Halo Assays used to create heat maps in mainFigures 3 & 4.

Supplement: Figure S1 [file mmc2.docx]
